# Information Support or Emotional Support? Social Support in Online Health Information Seeking among Chinese Older Adults

**DOI:** 10.3390/healthcare12171790

**Published:** 2024-09-07

**Authors:** Xiaowen Zhu, Chang Li

**Affiliations:** 1 School of Education, Suzhou University of Science and Technology, Suzhou 215009, China; 2 School of Architecture and Urban Planning, Suzhou University of Science and Technology, Suzhou 215009, China; lichang@usts.edu.cn

**Keywords:** online health information seeking, social support, online platforms, older adult healthcare, social network analysis

## Abstract

Online Health Information Seeking (OHIS) serves as an alternative form of social capital that can help older adults alleviate offline medical-related stress. This study collected and analyzed user interaction data from Patient-to-Doctor and Patient-to-Peer platforms and compared the roles of social support between them. Significant differences were identified in the dimensions of social support (information, emotional, and companion) on the Patient-to-Peer platforms compared with Patient-to-Doctor platforms (*p* < 0.05). The overall and core–core network density values for social support on Patient-to-Peer platforms were higher than those on Patient-to-Doctor platforms. Patient-to-Doctor interactions focused on information support, displaying a more centralized and efficient network with structural holes pertaining to treatment effects. By contrast, Patient-to-Peer interactions provided more emotional support, with a dispersed and redundant network containing structural holes related to individual information. Companion support was found to be weaker on both platforms. Additionally, digital literacy, surrogate seeking, and altruistic information significantly explained the variances between the two platforms (*p* < 0.01), with surrogate seeking playing a crucial role. These findings enhance our understanding of OHIS disparities among older adults and their surrogates, offering valuable insights for developing effective support systems and regulatory frameworks for health information platforms.

## 1. Introduction

In an era in which the population structure is rapidly shifting toward aging, the landscape of healthcare provision is also evolving swiftly. According to predictions, by 2050, China’s older population (aged 60 and above) will reach 500 million, accounting for one-third of the total population [1]. This demographic shift exerts considerable pressure on healthcare resources, both in terms of demand and costs. Simultaneously, the integration of the Internet with conventional healthcare resources has catalyzed the rise of online health information services. This service promotes scientific health management, fosters direct communication between healthcare providers and patients, simplifies medical procedures, and reduces the associated time and financial burdens.

During the lockdown period of the COVID-19 pandemic, a growing number of older adults turned to online platforms for health-related information and support. In this context, the significance of social support increased. These platforms enable them to access healthcare services, make health-related queries, and interact meaningfully with peers and healthcare providers. Access to online health information empowers them to comprehend professional healthcare details, maintain physical and mental well-being, engage in health-related decisions, and enhance their awareness of public health policies [2,3].

Different online health platforms provide varying social support to older adults in terms of content, audience, and influencing factors, yet these differences have not received sufficient attention from researchers. This study aimed to uncover nuanced disparities in social support dynamics during OHIS, highlighting unique social support traits and functional emphases across platforms. These insights will inform tailored support systems and regulatory frameworks for health information platforms catering to older adults in the digital era.

## 2. Literature Review

### 2.1. Older Adult OHIS

OHIS is the process by which individuals utilize online platforms to search for and retrieve health-related information, covering topics such as medical conditions, treatment effects, symptoms, healthcare providers, and lifestyle advice [4,5]. This process leverages diverse electronic resources like health websites, social media, and apps, enabling user engagement through queries, responses, and social connections. These platforms serve as essential archives of behavioral data in textual form, vital for studying OHIS patterns [6].

A variety of online health platforms have become vital channels for patients and their surrogates (e.g., families) seeking health information. Previous studies have predominantly centered on two platform types: Patient-to-Doctor and Patient-to-Peer. The former encompasses platforms like Health Tap, Mayo Clinic Connect, WebMD Community, and MedHelp, focusing on user queries, content characteristics, and the quality of the interactions [7,8,9]. Patient-to-Peer platforms, including PatientsLikeMe, HealthUnlocked, Reddit, and Inspire, are currently being explored for aspects such as user demographics, network properties, and the influence of opinion leaders in health discussions [10,11].

With the improvement of digital literacy of older adults, their engagement in OHIS garners academic attention. Studies indicate that the OHIS preferences of older adults primarily encompass healthy aging topics like disease awareness, medication details, medical procedures, diet and exercise guidelines, surrogate therapies, and end-of-life care [12]. Previous research has delineated two main categories of factors influencing older adult OHIS behavior: individual aspects, such as digital literacy, self-efficacy, and health conditions [13,14], as well as environmental aspects, including social norms, identity roles, platform usability, and the perceived relevance and accuracy of the information provided [2,15]. Moreover, age, gender, and health literacy have emerged as key determinants of OHIS conduct among older adults [16].

### 2.2. Social Support and Older Adult Health

Social support refers to the assistance, care, empathy, and motivation that an individual receives from their social circle (e.g., family, friends, peers, and community members), especially during challenging or difficult times. It includes information as well as emotional, instrumental, and evaluative support, which can aid individuals in managing stress, navigating adversities, fostering a sense of belonging, and enhancing overall well-being [17]. Since the late 1960s, the concept of social support has been pivotal in health-related research, emerging as a key determinant of physical and mental health outcomes. Interdisciplinary efforts by epidemiologists, health psychologists, sociologists, and other experts have strengthened the assessment and comprehension of social support’s impact [18]. Notable models like Berkman and Glass’s framework examining the role of social networks and support in health outcomes [19], along with tools like the ENRICHD Social Support Inventory focusing on information, emotional, and practical support dimensions [20,21], have enriched this understanding. Numerous studies affirm that powerful social connections serve as protective factors against health deterioration and promote the physical and mental well-being of the older population [22].

The advancement of ICTs has remarkedly transformed traditional forms of social support, giving rise to online platforms as vital channels for supporting older adults facing chronic illnesses, life-threatening diseases, or dependency issues. Scholars have redefined social support within the digital context as not just surrogate support but as an essential integration tool for informational and medical resources [23,24]. These online structures allow for the dissemination of crucial health information and the creation of support networks unbounded by geographical or physical constraints.

Existing research has identified three primary types of social support in health platforms: information support empowers users with knowledge, emotional support is fostered through community interactions, and practical support guides real-world actions. Online health platforms and social media groups serve critical roles in this dynamic, providing older adults access to a broader spectrum of social support beyond offline healthcare settings, where environmental stressors (e.g., institutional environments) could impede their experiences and outcomes [25,26]. Studies have also outlined barriers that older adults encounter in accessing social support for online health, including limited digital and health literacy, alongside psychological obstacles.

### 2.3. Objectives

While existing studies have extensively examined the usage patterns and benefits of OHIS on Patient-to-Doctor and Patient-to-Peer platforms, studies that quantify and compare the distinctiveness and influencing factors of these platforms have been limited by technical constraints. Moreover, current social support investigations concerning OHIS among older adults have neglected to analyze the social support emphasis and key influencing factors specific to Patient-to-Doctor and Patient-to-Peer platforms. These research gaps necessitate further exploration. Taking these into consideration, this study aims to empirically quantify the social support characteristics of Patient-to-Doctor and Patient-to-Peer platforms in OHIS among older adults using social network analysis and pertinent statistical methodologies. This exploration seeks to address the following questions: (a) What variations exist in social support dimensions between the utilization of Patient-to-Doctor and Patient-to-Peer platforms? (b) What distinctions exist in motivational and influencing factors driving the use of Patient-to-Doctor and Patient-to-Peer platforms for OHIS to access social support?

## 3. Materials and Methods

### 3.1. Data Resource

The data were obtained from open-access online health information platforms, specifically Patient-to-Doctor (https://www.haodf.com/ [accessed on 14 December 2023]) and Patient-to-Peer (https://tieba.baidu.com/ [accessed on 18 December 2023]). Patient-to-Doctor serves as a proprietary platform facilitating Q&A exchanges between users and healthcare professionals, while Patient-to-Peer functions as an open platform allowing users to post, retweet, and comment on an original post. 

The data were subject to inclusive criteria requiring content in text format and relevance to health topics of older adults. They were collected in a transparent way, primarily through voluntary user contributions (between January 2016 and December 2023). Platforms utilized anonymization techniques for key identifiable information, ensuring no influence on the study outcomes. By the end of 2023, a total of 1595 full-text Q&A exchanges for Patient-to-Doctor platforms and 229 original posts for Patient-to-Peer platforms (including 1527 times of posting, retweeting, commenting, and mentioning) were downloaded as original data using ActivePython version 2.7.12.

### 3.2. Coding Framework

Drawing from prior research, we developed a new coding framework wherein social support was reconceptualized and reorganized into three dimensions: information, emotional, and companion support. These dimensions were defined as follows: information support entailed soliciting knowledge, expertise, or information on specific topics or themes; emotional support involved seeking emotional feedback or understanding, with a focus on perceptions and emotions; and companion support encompassed connecting, interacting, or motivating other users or groups, typically centered around tangible activities or relationships.

**Figure 1:**
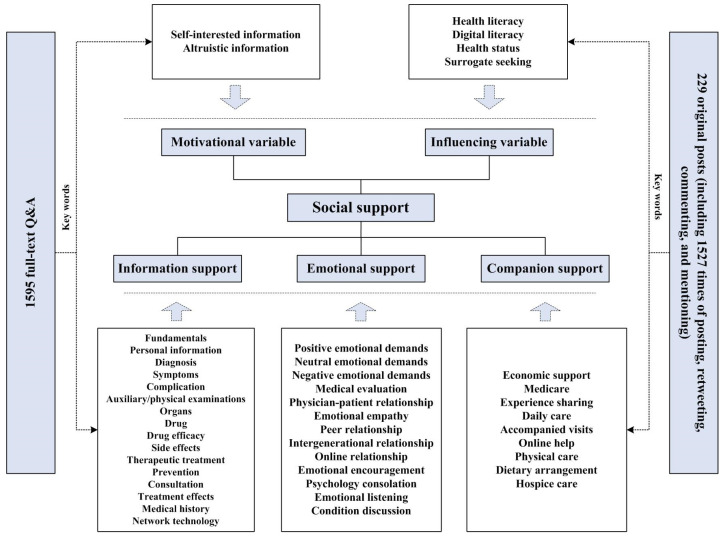
The coding framework.

In the data preprocessing phase, we conducted text segmentation using NLPIR (Natural Language Processing and Information Retrieval). NLPIR-ICTCLAS is a software designed for natural language processing and information retrieval tasks. Through NLPIR, we were able to effectively remove punctuation marks (e.g., question marks and exclamation marks), correct textual and grammatical errors, eliminate inappropriate spaces, and filter out irrelevant information. These capabilities ensured that our textual data was clean and structured, facilitating more accurate and efficient analysis. NLPIR is widely used in applications such as text mining, automatic summarization, sentiment analysis, and information extraction [27]. Subsequently, we extracted keywords from the generated content and performed topic clustering (Table 1, Figure 1). For instance, on a Patient-to-Doctor platform, a doctor responded: “Your *coronary CT scan* ^①^ shows *moderate to severe narrowing of the coronary arteries* ^②^, indicating the need for an *angiography* ^③^.” The keywords ① and ③ belonged to the factor of auxiliary/physical examinations, while the keyword ② fell under the factor of diagnosis, categorizing both under information support. On a Patient-to-Peer platform, a peer commented: “Try to be more *optimistic* ^④^. *Positive attitude* ^⑤^ is crucial. Like me, I have been diagnosed with *coronary heart disease and atrial fibrillation* ^⑥^, but I live as if *nothing’s wrong* ^⑦^.” The keywords ④⑤⑦ were categorized under the factor of emotional encouragement, whereas the keyword ⑥ was classified under diagnosis. The former pertained to emotional support, while the latter related to information support.

Adhering to the triangulation principle, three skilled researchers were invited for paid work on coding [28]. Before formal coding, we organized three pretests of coding and three workshops, adjusting the initial social support coding table accordingly. Once Cohen’s α values between the three researchers for the dimensions reached 0.75, indicating the result passed intercoder reliability tests, we began the formal coding process by Nvivo12 independently. The network structure of the study was assessed by metrics of network size, density, core-periphery class membership, degree centrality, closeness centrality, structural holes, and reciprocity. Tie strength, as introduced by Granovetter (1973), refers to the closeness and intensity of a social relationship [29]. It is assessed by measuring the frequency and reciprocity of interactions between users across both platforms. 

### 3.3. Social Network Analysis

Social network analysis encompasses a set of norms and methods for analyzing the structure of relationships and attributes within networks based on data among actors. Algebraic models and graphical theory tools are used to quantify p and degrees and expand on each actor’s position and influence within the network. Our study shows that older adults, doctors, peers, and surrogates engage in interactions on platforms, forming complex relationships and shaping a network structure. Social network data were collected through content analysis, capturing the dimensions of sought and provided support, as well as the frequency of user interaction [30]. Automatic web page crawling was carried out using Python. Data processing and social network analysis were conducted by Nvivo 12 and SPSS 24, while graphic construction was executed by UCINET 6 and NetDraw. Commonly used indicators in social network analysis include network density, degree centrality, closeness centrality, and structural holes.

Network density stands as a key metric for identifying the overall network status of both Patient-to-Doctor and Patient-to-Peer platforms [31]. The formula for network density is outlined in Equation (1), where *L* and *N* represent the number of links and nodes within the network, respectively. A higher density value indicates a more interconnected network of nodes. Core-periphery class membership conceptually divides a network into a tightly interconnected core group and a more loosely linked periphery group. These two metrics collectively measure the overall structure of a network.

(1) $$D=\frac{L}{N\times \left(N-1\right)\times 0.5}$$

Degree centrality is a metric used to evaluate the prominence of nodes in a network based on their out-degree or in-degree values, illustrating a node’s influence within the network. It is calculated using Equation (2), where *k_i_* represents the number of edges adjacent to node *i*, and *N* denotes the total number of nodes in the network. Typically, a higher value of degree centrality signifies greater importance within the network. In this study, the data is represented by a symmetric matrix, the in-degree and out-degree centrality values are the same in this context. Therefore, we used in-degree to conduct the statistical analyses.

(2) $${DC}_{i}=\frac{k_{i}}{N-1}$$

Closeness centrality measures the average shortest distance from a node to all other nodes in the network. The equations for calculating closeness centrality are outlined in (3) and (4). *d_i_* represents the average distance from node *i* to the other nodes, with the reciprocal of *d_i_* representing the value of closeness centrality. A higher value of closeness centrality for a node signifies a central position within the network.

(3) $$d_{i}=\frac{1}{N-1}\sum_{j=1}^{N}d_{ij}$$

(4) $$CC_{i}=\frac{1}{d_{i}}$$

Structural holes refer to the gaps between node clusters lacking direct and indirect redundant relationships but containing complementary information, resembling holes in a network. According to Burt (1992), the nodes occupying these structural holes have the potential to control and facilitate the flow of information across the network [32]. Effective size and constraint are the primary indices for quantifying the control that structural hole nodes have over these relationships. Effective size measures the number of non-redundant contacts a node has, indicating its potential to access diverse information. Conversely, constraint quantifies the extent to which a node’s connections are connected to each other, thereby limiting its brokerage potential. In general, a node with a larger effective size and smaller constraint is identified as occupying a structural hole, thereby serving as a crucial bridge between node clusters.

## 4. Results

### 4.1. The Similarities and Differences of Social Support in OHIS on Two Platforms

**Figure 2:**
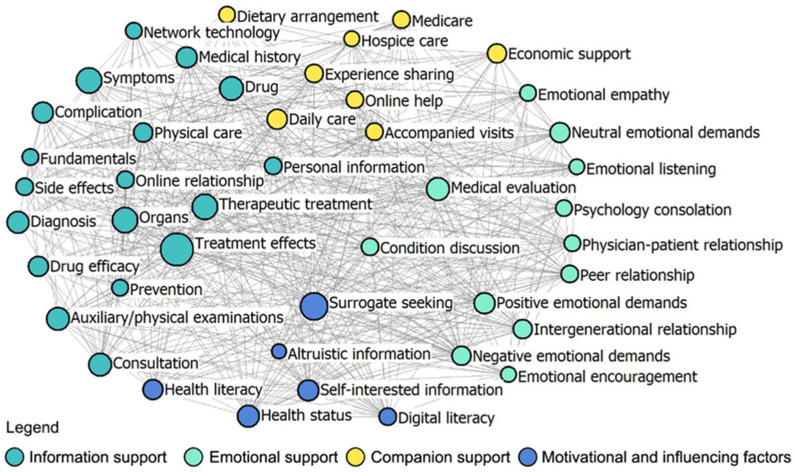
An overall network of Patient-to-Doctor platforms.

**Figure 3:**
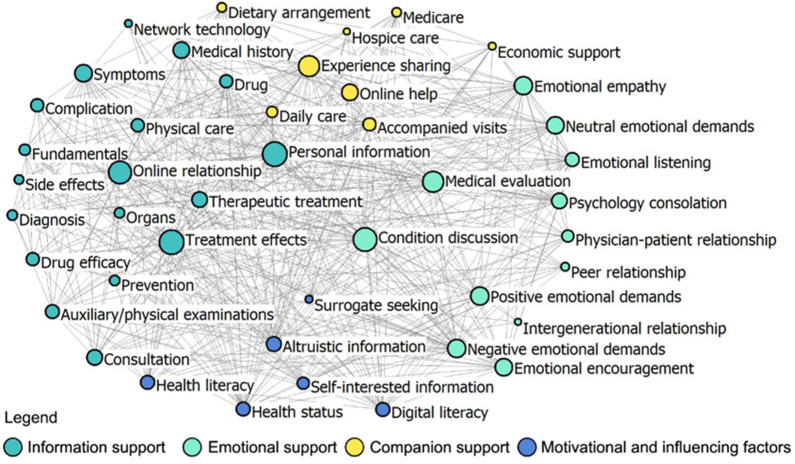
An overall network of Patient-to-Peer platforms.

Network density and core-periphery class membership are utilized to evaluate the degree of connectivity saturation and centrality surrounding specific nodes within the social support network structure of the platforms. The paired sample t-test results demonstrated that the overall network density in Patient-to-Doctor platforms (value = 13.86) is significantly lower than that in Patient-to-Peer platforms (value = 22.77) (*p* < 0.01). Furthermore, the analysis of core-periphery class membership revealed distinctions in the density values of core–core (56.96: 24.57), periphery–periphery (8.47:0), and core–periphery (13.43: 4.64) in Patient-to-Doctor and Patient-to-Peer platforms (Table 2). Visual representations of network density and core-periphery class membership are provided in Figure 2 and Figure 3, highlighting distinctions in the network overall structure of the two platforms. 

### 4.2. Network Nodes

**Figure A1:**
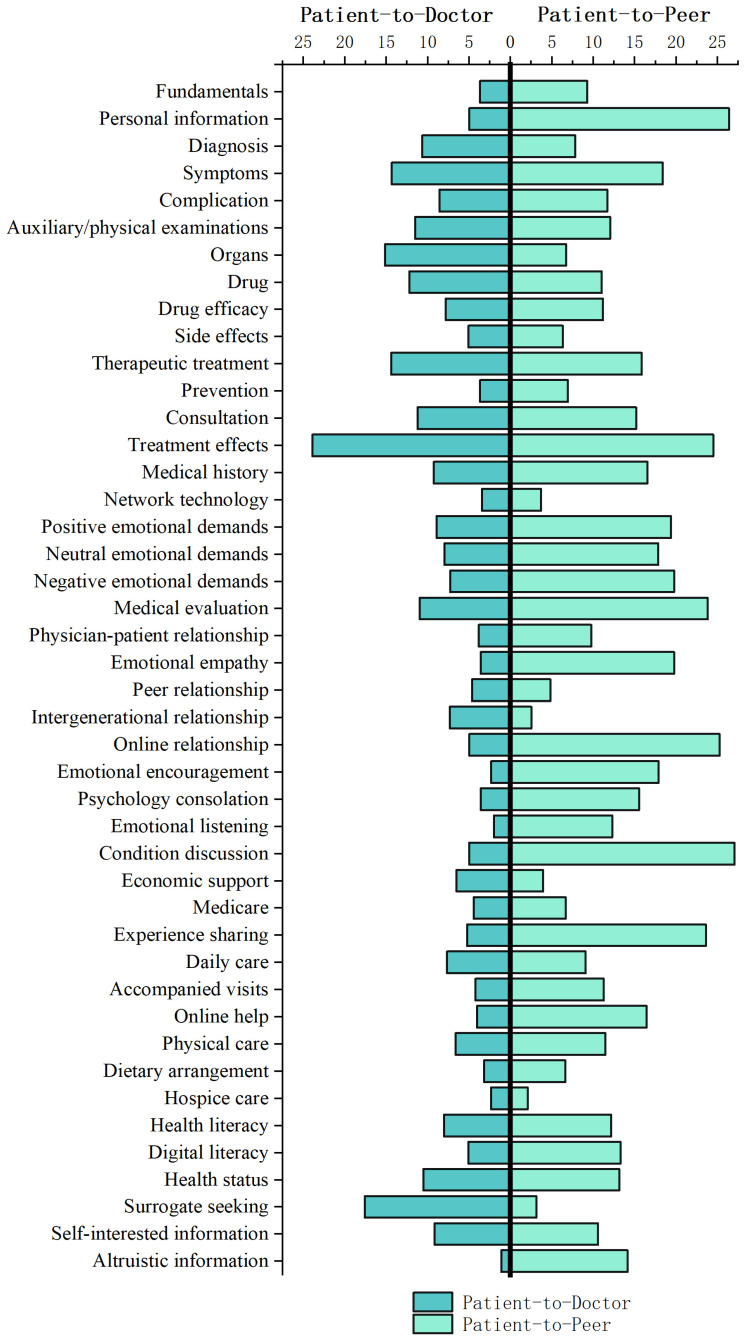
The difference in degree centrality between the two platforms.

**Figure A2:**
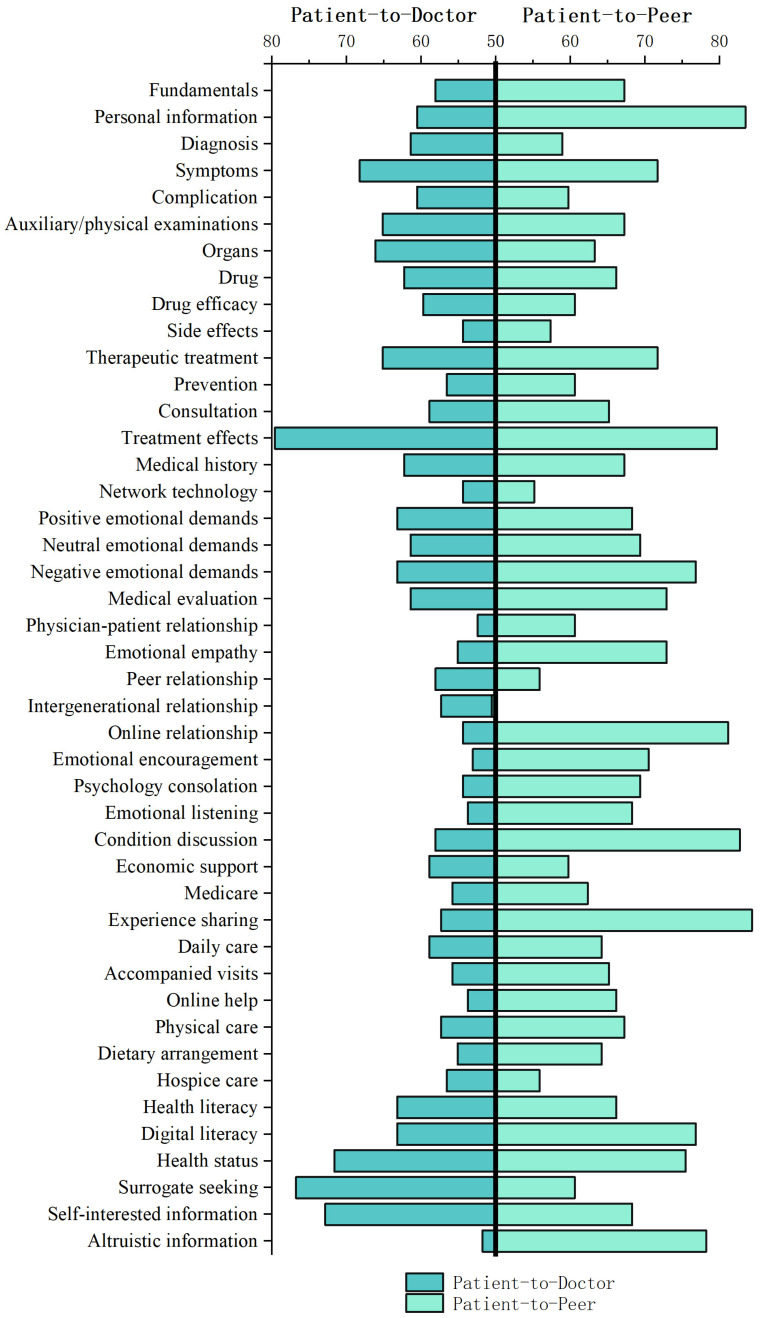
The difference in closeness centrality between the two platforms.

Network node comparison was used to explain social support differences in OHIS for older adults in China across Patient-to-Doctor and Patient-to-Peer platforms. Paired sample t-tests conducted for degree centrality and closeness centrality unveiled significant discrepancies between two platforms across the overall network and three dimensions (*p* < 0.05), as depicted in Table 3. Specifically, in terms of degree centrality metrics, disparities between Patient-to-Doctor and Patient-to-Peer platforms were observable in factors such as emotional encouragement, online relationship, and emotional empathy (Figure A1); similarly, variations in closeness centrality metrics are noted in factors like online relationship, experience sharing, and condition discussion (Figure A2). These findings indicate that Patient-to-Peer platforms provide stronger emotional support and foster more significant online relationships and emotional empathy compared with Patient-to-Doctor platforms. The higher degree centrality metrics for emotional encouragement and online relationships on Patient-to-Peer platforms suggest that older adults are more engaged in these peer networks. The variations in closeness centrality metrics further support this, showing that older adults on Patient-to-Peer platforms benefit from more experience sharing and condition discussions, enhancing their social support network. 

**Figure A3:**
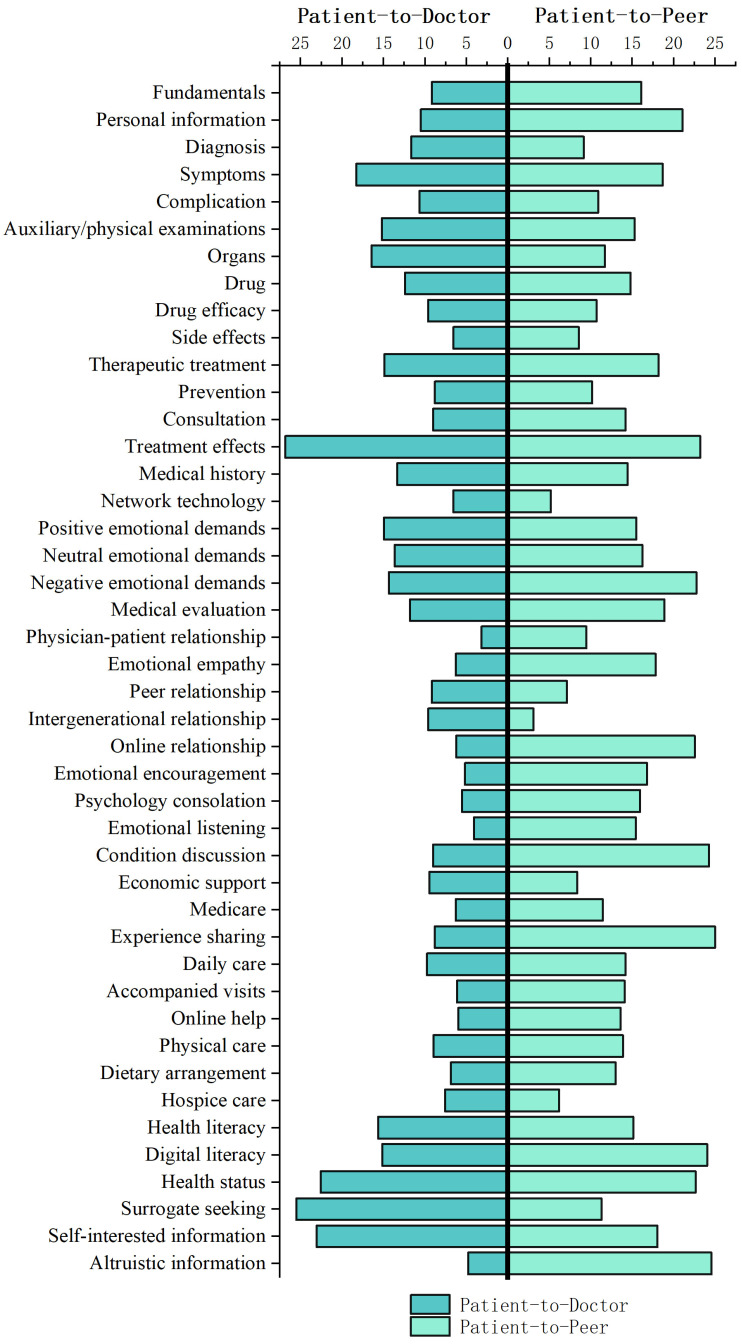
The difference in effective size of structural holes between the two platforms.

**Figure A4:**
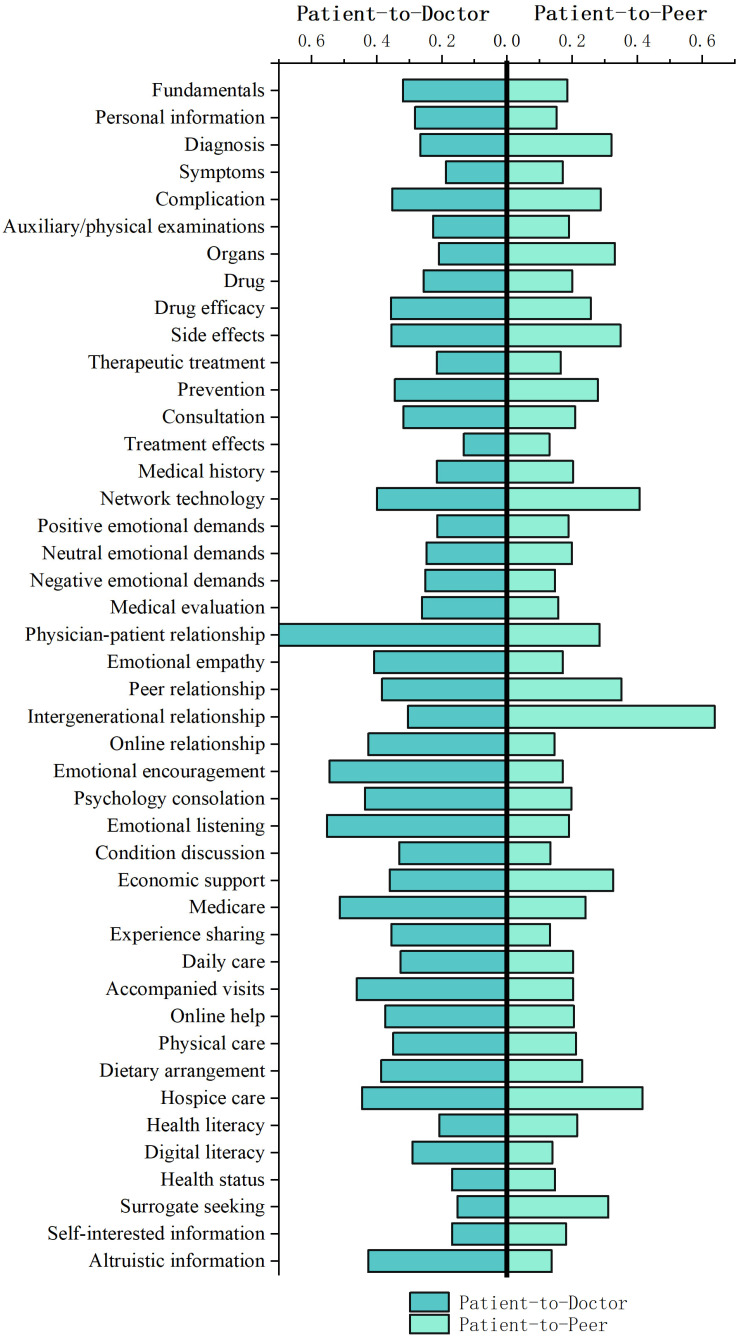
The difference in constraint value of structural holes between the two platforms.

Furthermore, the results highlight that the structural holes for Patient-to-Doctor platforms pertain to treatment effects (effective size = 26.84, constraint value = 0.13), whereas for Patient-to-Peer platforms, it is personal information (effective size = 21.08, constraint value = 0.15), as illustrated in Figure A3 and Figure A4. The analysis of structural holes reveals distinct network structures between the two platforms. Patient-to-Doctor platforms are more centralized around treatment effects, indicating a focus on disseminating treatment-related information efficiently. By contrast, Patient-to-Peer platforms are more centered around personal information, reflecting a dispersed network that supports broader and more varied interactions.

### 4.3. Motivational and Influencing Variables for Older Adults in OHIS on Two Platforms

The analysis of motivational factors based on Pearson correlation tests and Paired t-tests revealed a significant correlation for self-interested information among older adults engaging in OHIS between the Patient-to-Doctor and Patient-to-Peer platforms (r = 0.377, *p* < 0.05), while a notable difference was observed for altruistic information (*p* < 0.01). These findings indicate that older adults’ engagement in OHIS is driven by both self-interested and altruistic motivations, with significant differences in how altruistic information is prioritized between Patient-to-Doctor and Patient-to-Peer platforms.

Furthermore, the examination of influencing factors in OHIS demonstrates a substantial correlation in health literacy between the two platforms (r = 0.509, *p* < 0.01). By contrast, factors such as digital literacy and surrogate seeking exhibit significant disparities (*p* < 0.01). These findings suggest that older adults on Patient-to-Peer platforms may exhibit higher digital literacy, facilitating their engagement and interaction in these environments. Similarly, the pronounced difference in surrogate seeking indicates that older adults might rely more on surrogates in Patient-to-Doctor settings to navigate health information. There was no statistically significant distinction in the factor of health status (Table 4).

## 5. Discussion

This study introduces a research methodology that integrates information coding with social network analysis to compare the social support characteristics and key factors among older adults engaging in OHIS on the Patient-to-Doctor and Patient-to-Peer platforms. Diverging from prior research that predominantly focuses on user behaviors in OHIS, this study employed a set of metrics to contrast the characteristics between the two platforms, including network density, degree centrality, closeness centrality, and structural holes. A notable achievement of this study is the provision of visual representations and empirical findings elucidating the traits of the two platforms, thus enriching the methodological aspect, which serves as the main contribution of this study. This research methodology exhibits versatility for application in other fields, attributed to its broad applicability and robustness.

Some findings of this study align with prior research, showing OHIS as a social capital alternative to offline healthcare [33]. Additionally, it reinforces that information sharing and emotional interaction are key aspects of social support in OHIS [25], along with highlighting the negative impact of limited digital literacy and health literacy on older adults’ OHIS behaviors [20,34]. The findings on health conditions and self-interested motivations similarly corroborate prior findings related to offline health information seeking among older adults [16,22].

The most significant contribution of this study lies in breaking through the previous separate studies on the Patient-to-Doctor and Patient-to-Peer platforms, as well as addressing the lack of older subjects in the study. The study reveals that the overall network density and core–core network density of social support in OHIS on the Patient-to-Doctor platform are higher than those on the Patient-to-Peer platform. This indicates that the social support network for older adults on the Patient-to-Doctor platform is more centralized and efficient, while that on another platform is more dispersed and redundant. Further analysis based on the degree centrality and closeness centrality of network nodes reinforces this conclusion. On one hand, social support within the Patient-to-Doctor platform centers around information support, with users (comprising older patients and their surrogates) and doctors primarily involved in effective communication concerning the topic of “treatment effects”, while direct interaction among users is infrequent. This undervalues the significance of the Patient-to-Doctor platform in delivering succinct, timely, expert, and tailored information to address users’ health concerns. On the other hand, emotional support emerges as the key social support dimension within the Patient-to-Peer platform. OHIS activities are predominantly led by older patients themselves, leading to the formation of a patient community actively involved in seeking health information and sharing emotional experiences. According to Social Exchange Theory, human behavior does not always aim to maximize benefits but rather seeks to attain the most suitable advantages in social interactions. Thus, social interaction involves not only the exchange of information and money but also encompasses elements such as praise, self-esteem, love, and emotion [35]. Additionally, compared with information and emotional support, the proportion of companion support, indicated by fewer key nodes, is low on both platforms. While online support facilitates meaningful peer-to-peer connections among users, it cannot fully replace the significance of companionship. The essence of companionship is rooted in real-world interactions, which are essential and irreplaceable, especially in the context of caring for and supporting older adults who may be in fragile physical conditions. 

Another notable finding of this study is the distinction in intermediary factors (structural holes) of the social support within the two platforms. For the Patient-to-Doctor platform, the structural hole of treatment effects stands out. As highlighted earlier, the objective of Patient-to-Doctor interactions is clear and direct: to acquire effective information and address health-related concerns. Users participate in discussions with doctors on treatment effects, leading to the formation of a densely interconnected network centered around this aspect. Conversely, for the Patient-to-Peer platform, the structural hole is related to user information. The successful conveyance of information hinges on detailed descriptions of personal attributes, individual requirements, and specific backgrounds. With online communication increasingly becoming a vital component of social support, it enables individuals to cultivate social connections with others through remote technology [36]. Older users find and filter peers by accurately describing their information, thus forming a “small world”. As emphasized by the Peer Effect Theory, individuals with similar attributes are more likely to establish social relationships [37]. Older users who share similar health conditions or possess comparable levels of health literacy, for instance, are more inclined to establish trust with one another [38], adopt each other’s advice, and share health attitudes [39,40]. This peer effect further deepens the emotional support mentioned earlier. 

The study also unexpectedly discovered the significant role of surrogate seeking on Patient-to-Doctor platforms. Statistics indicate that intergenerational members are the main participants in surrogate seeking. This finding aligns with previous research, indicating that social support can promote health behaviors and facilitate the achievement of personal goals. Family relationships, especially intergenerational support, play a crucial role in encouraging older adults to utilize the Internet to access necessary health information [41,42]. Older adults tend to place trust in their family members, and the act of surrogate seeking enhances their capacity to obtain and utilize health information, subsequently impacting their perceived self-efficacy and influencing their health-related behaviors and decisions. This behavior also prompts their family members to provide improved support in assisting the older adults with managing health conditions, compensating for any gaps in information and health knowledge among the older adults. Additionally, disparities in usability also influence their preference for surrogate seeking. Research suggests that “ease of navigation” ranks among the fundamental attributes of an age-friendly website [43]. In practical terms, the navigation of the Patient-to-Doctor platform is more complex, with communication topics and content geared toward a higher level of professionalism. This complexity may present greater psychological and technological barriers for older users with lower e-health literacy, rendering it challenging for them to navigate the platform easily. Conversely, the Patient-to-Peer platform is designed to be more age-friendly, as reflected in its user-friendly webpage interface and high readability.

## 6. Limitations

While the analysis, based on the information coding of OHIS and social network analysis, contributes to an objective comparison of social support variances between Patient-to-Doctor and Patient-to-Peer platforms, there are still limitations within this study. Firstly, the research focuses solely on Chinese OHIS platforms, neglecting an extensive analysis of international platforms. Future studies should include evaluations of a wider range of OHIS platforms to deepen understanding. Secondly, the data used in the study are from open-access platforms, lacking details on individuals’ social identities and preferences (including age, gender, education, income, health information, and emotional inclinations) due to digital privacy constraints. These aspects are critical and warrant further exploration. Thirdly, OHIS represents only a fragment of the sources of social support available to older adults, traditional face-to-face interactions continue to play a significant role in the social support networks of older adults. Future research should try to integrate or compare these modes and present a more comprehensive picture of social support.

## 7. Conclusions

Social support has been identified as one of the most crucial facilitating conditions that positively impact older adults’ OHIS behaviors [44]. Our study extracted three social support dimensions and two explanatory variables from 1595 full-text Q&A exchanges from the Patient-to-Doctor platform and 229 original posts from the Patient-to-Peer platform. Through quantitative analysis using social network methods, we revealed the social support of older adults’ OHIS behaviors. 

The study found that the values of overall network density of the Patient-to-Doctor and Patient-to-Peer platforms were 13.86 and 22.77, respectively, with core–core network densities of 56.96 and 24.57. This indicates that the social support on Patient-to-Doctor platforms is more centralized and efficient. Regarding network nodes, the paired sample t-test of degree centrality and closeness centrality showed significant differences (*p* < 0.05) in both platforms’ overall network and the three dimensions. Patient-to-Doctor platforms lean toward information support, with treatment effects as its structural hole, whereas Patient-to-Peer platforms lean toward emotional support with personal information as its structural hole. Specifically, core factors such as emotional encouragement, online relationship, and emotional empathy on both platforms show differences in the metric of degree centrality, while core factors like online relationship, experience sharing, and condition discussion show differences in the metric of closeness centrality. Moreover, in analyzing the motivational and influencing factors that distinguish between the two platforms, the study uncovered that digital literacy, surrogate seeking, and altruistic information play significant roles in explaining the differences between the platforms (*p* < 0.01). The correlations observed between the two platforms in the core factors of self-interested information and health literacy were also found to be significant (*p* < 0.05). These insights enhance our understanding of improving platform design, developing policies and regulations, and supporting systems for OHIS. By addressing these areas, we can create a more inclusive environment that recognizes and adapts to the unique needs of older adults in the digital age. This approach not only improves their health information-seeking experiences but also contributes to their overall well-being and social inclusion.

## Figures and Tables

**Table 1:** Word cloud of keywords.

	Patient-to-Doctor	Patient-to-Peer
Information support	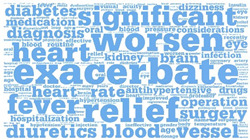	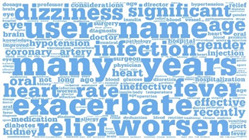
Emotional support	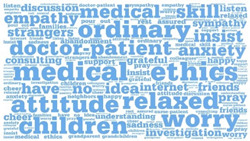	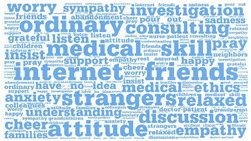
Companion support	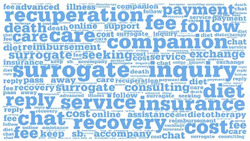	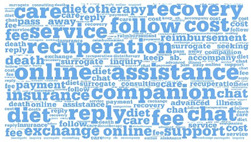

**Table 2:** The statistical results of core–periphery class membership.

	The Density Matrix			Fitness Value
	Core–Core	Core–Periphery	Periphery–Periphery	
Patient-to-Doctor	56.955	13.427	8.468	0.542
Patient-to-Peer	24.57	4.64	0.000	0.026

**Table 3:** Results of the paired t-test analysis on degree centrality and closeness centrality of Patient-to-Doctor and Patient-to-Peer platforms.

	Degree Centrality		Closeness Centrality	
	*t*	*p*	*t*	*p*
Overall	−4.951	<0.001	−5.103	<0.001
Information support	−2.081	0.050	−2.940	0.010
Emotional support	−5.352	<0.001	−4.332	<0.001
Companion support	−2.370	0.045	−3.292	0.011

**Table 4:** The similarities and differences in the motivational and influencing variables of older adults in OHIS on the two platforms.

Factor	Pearson Correlation Test		Paired *t*-Test	
	*r*	*p*	*t*	*p*
Self-interested information	0.377	0.018	−1.835	0.074
Altruistic information	0.025	0.879	−7.274	<0.001
Health literacy	0.509	<0.001	−1.343	0.187
Digital literacy	−0.284	0.080	−2.814	0.008
Health status	−0.173	0.292	−0.548	0.587
Surrogate seeking	0.087	0.599	4.348	<0.001

## Data Availability

Raw data supporting the conclusions of this article will be made available by the authors upon request.
